# Adaptation of fear of missing out scale (FoMOs): Turkish version validity and reliability study

**DOI:** 10.1186/s41155-019-0117-4

**Published:** 2019-01-22

**Authors:** Gurhan Can, Seydi Ahmet Satici

**Affiliations:** 1 Department of Psychological Counseling and Guidance, Hasan Kalyoncu University, Faculty of Education, Gaziantep, Turkey; 2 Department of Psychological Counseling and Guidance, Artvin Coruh University, Faculty of Education, Artvin, Turkey

**Keywords:** Fearing of missing out, Social media, Psychometric properties, Scale adaptation

## Abstract

**Background:**

The aim of this study is to examine psychometric properties of the Turkish version of the fear of missing out scale (FoMOs) on three different study groups.

**Method:**

We conducted the construct validity of the Turkish FoMOs with confirmatory factor analysis, measurement invariance (study I; *n* = 354), and concurrent validity (study II; *n* = 371). We also evaluated the reliability of the Turkish FoMOs (study III; *n* = 61) using test-retest and Cronbach alpha reliability.

**Results:**

In study I, the confirmatory factor analysis revealed that the one-dimensional structure of the Turkish version of the FoMOs was verified. The results of measurement analysis depending on the sample of study I demonstrated that configural and metric invariances were established across Facebook and other social media users. The Cronbach alpha values calculated from the samples of study I (α = .79) and study II (α = .78) indicated that internal consistency of the scale was at the acceptable level. Lastly, test-retest reliability of the scale was found as .86 from the study III.

**Conclusion:**

Overall findings indicated that the psychometric properties of the Turkish version of FoMO scale were satisfactory to measure the FoMO in a wide range of ages in the Turkish context.

With the spread of smartphones and other mobile devices in recent years, the number of social media users in all countries has been increasing rapidly. The latest Global Digital Statshot report has stated that the number of social media users around the world exceeded three billion (Kemp, 2017). The same report also reveals that Turkey ranks ninth among 238 countries with 48 million people (60% of the population) reporting some social media use. Although social media gives us instant access to all news, information, documents, and the opportunity to share audio and videos, it also has a darker side. One of them is that unconscious and excessive use of social media can change the daily habits of individuals (King, Valença, Silva, Baczynski, Carvalho, & Nardi, 2013; Liftiah, Dahriyanto, & Tresnawati, 2016). Moreover, social media addiction can cause long-term damages to the individual’s emotions, behaviors, and relationships (Rao, 2017). On the other hand, the intensive use of social media induces behavioral changes of its users (Liftiah et al., 2016) and “may also trigger the individual’s fear of missing out (FoMO)” (Wegmann, Oberst, Stodt, & Brand, 2017, p .33).

FoMO is a kind of social anxiety (Dossey, 2014) caused by worries that others may have more satisfying lives than themselves. FoMO is defined as “a pervasive apprehension that others might be having rewarding experiences from which one is absent” and “a desire to stay continually connected with what others are doing” (Przybylski, Murayama, DeHaan, & Gladwell, 2013, p. 1841). According to another definition suggested by JWT Intelligence (2011), FoMO is “the uneasy and sometimes all-consuming feeling that you’re missing out—that your peers are doing, in the know about, or in possession of more or something better than you”. Przybylski et al. (2013) who associate the FoMO with the self-determination theory (SDT) (Deci & Ryan, 1985) argued that this is caused by the individual’s unsatisfied basic psychological needs such as *autonomy*, *competenc*e, and *relatedness.* They also stated that FoMO can be associated with the *esteem* and *social needs* explained in the human motivation theory (Maslow, 1943). Depending on their findings, Przybylski et al. (2013) suggested that “Fomo can be understood as a self-regulatory state arising from situational or long-term perception that one’s needs are not being met”. On the other hand, the uses and gratifications theory (Blumler & Katz, 1974), which focuses on why and how people use the social media, claims that people actively choose and use the social media because they would like to satisfy their specific needs such as social interaction, entertainment, information seeking, and sharing (Dolan, Conduit, Fahy, & Goodman, 2016; McQuail, 2010).

Previous research findings on the FoMO have indicated that FoMO is positively associated with negative social and emotional experiences, such as boredom, loneliness, irritability, and inadequacy (Abel, Buff, & Burr, 2016; Edwards, 2017; Lampe, Ellison, & Steinfield, 2007). Some other researchers have also indicated that FOMO is negatively associated with positive traits such as well-being, overall mood, and life satisfaction (Burke, Marlow, & Lento, 2010; Przybylski et al., 2013; Wortham, 2011).

As mentioned above, previous research has pointed out that the factors affecting FoMO and the variables affected by FoMO have been studied extensively. For example, FoMO has been associated with intensive social media use, such as social networking addiction (Blackwell, Leaman, Tramposch, Osborne, & Liss, 2017; Kuss & Griffiths, 2017; Tomczyk & Selmanagic-Lizde, 2018; Wang et al., 2018), the amount of stress experienced when using social networking sites (Beyens, Frison, & Eggermont, 2016), use of mobile phones while driving/learning (Przybylski et al., 2013), social media fatigue (Bright & Logan, 2018), decreased self-esteem (Buglass, Binder, Betts, & Underwood, 2017), poor sleep (Adams et al., 2016), college maladjustment (Alt, 2018), smartphone addiction (Wolniewicz, Tiamiyu, Weeks, & Elhai, 2018), neuroticism (Blackwell et al., 2017), depression (Elhai, Levine, Dvorak, & Hall, 2016), anxiety (Blackwell et al., 2017; Elhai et al., 2016), and negative alcohol-related consequences (Riordan, Flett, Hunter, Scarf, & Conner, 2015).

In the FoMO literature, FoMO scales have been developed by Przybylski et al. (2013), Abel et al. (2016), Metin, Pehlivan, and Tarhan (2017), and Riordan et al. (2018). The most common and popular one of them used worldwide is the single factor FoMO scale (henceforth the FoMOs) (Przybylski et al., 2013), which is relatively simple and convenient as it contains only ten items.

The FoMOs has been adapted previously to Turkish (Gökler, Aydın, Ünal, & Metintaş, 2016), Arabic (Al-Menayes, 2016), Spanish (Gil, Oberst, Del Valle, & Chamarro, 2015), and English (Perrone, 2013; for American adolescents). The Arabic version of the FoMOs showed a two-factor structure with .82 and .72 Cronbach alpha values while the Turkish, Spanish, and English versions supported the original one-factor structure with .81, .85, and .93 Cronbach alpha values respectively. Although the first adaptation of the FoMOs in Turkish was accomplished (Gökler et al., 2016) prior to the present study, the authors conducted their study just on a group consisting of only 200 university students with an average age of 21.4. Thus, this meant that the first Turkish version of the FoMOs could be valid only for young university students and that a new version of the scale is needed for the justification of the validity of research to be undertaken on older target groups. Therefore, the purpose of this study was to carry out and report a series of the FoMOs’ adaptation studies on three different study groups in Turkey. Therefore, the purpose of this study was to carry out and report a series of the FoMOs’ adaptation studies on three different study groups in Turkey.

The current scale adaptation study was carried out in three different study groups. In the first step of the first study, the original English version of the FoMOs was translated into Turkish. In the second step, the structure validity and the internal consistency of the Turkish version of the FoMOs was examined (study I, *n* = 354). In the second study, the concurrent validity of the Turkish version of the FoMOs was examined (study II, *n* = 371). In the third study, the test-retest reliability of the Turkish version of FoMOs was examined (study III, *n* = 60).

## Study I

### Purpose

Study I aimed to translate the FoMOs into Turkish and examine the construct validity of the Turkish version of the FoMOs.

### Method

#### Participants

The participants were 152 male (42.9%) and 202 female (57.1%) social media users at the ages ranging from 15 to 72 (*M* = 32.97, *SD* = 13.04), 56.8% of whom were employees, 10.5 retired, and 32.8 students. The participants’ preferred social media platforms were Facebook (59.9%), Whatsapp (24%), Twitter (8.5%), and Instagram (5.6%).

The answer to the question of the most important reason why participants use social media demonstrated that 53.4% of them used the social media to keep track of social information, 26.3 to communicate with friends, 20.3 to share ideas, opinions, and audio/visual material such as videos and pictures.

#### Data collection process

In order to achieve the best possible results for the psychometric properties of the scale (Buhrmester, Kwang, & Gosling, 2011), an on-line surveying method was used during the data collection process instead of the conventional methods. All scales and personal information form (PIF) were transferred to an online environment via Google Forms. The authors made this Google Forms available to the public through social media channels. Therefore, the study was carried out with volunteer participants reaching the Form and not receiving any incentives.

### Measures

#### Fear of missing out scale (FoMOs; Przybylski et al., 2013)

The scale is a one-factor ten-item (e.g., I get anxious when I do not know what my friends are up to) self-report measurement. Each item is rated on a 5-point Likert scale (1 = *Not at all true* to 5 = *Absolutely true*). The total scores of the scale range between 10 and 50, where higher scores indicate a higher level of fear of missing out. The Cronbach α coefficient of the original version of the scale is .90.

#### Personal information form

PIF was prepared by the researchers. The PIF has consisted of demographic questions such as age, gender, personal status (employees, retired, students), and also social media questions such as the most preferred social media platforms, reasons of using social media.

### Procedures

The original version of FoMOs was translated into Turkish following the linguistic equivalency procedures described by Brislin (1970, 1980). The data were examined using LISREL 8.53 (Jöreskog & Sörbom, 1993) for the structure validity of the Turkish FoMOs. A confirmatory factor analysis was performed using maximum likelihood on the ten-observed items of the Turkish FoMOs to provide empirical-based evidence for determining whether the Turkish version of the FoMOs would yield similar structure to the original version of the FoMOs. The plausibility of differing factor structures associated with gender was also tested. The goodness-of-fit of the models was evaluated by Chi-square (χ^2^), Chi-square to the degree of freedom ratio (χ^2^/df), comparative fit index (CFI), goodness-of-fit index (GFI), standardized root-mean-square residual (SRMR), and root mean square error of approximation (RMSEA).

### Results

The data were checked for normality by kurtosis and skewness. They were considered to be normal because the skewness value was .22 (between − 2 and + 2), and the kurtosis value was − 79 (between − 2 and + 2) for all variables.

**Fig. 1:**
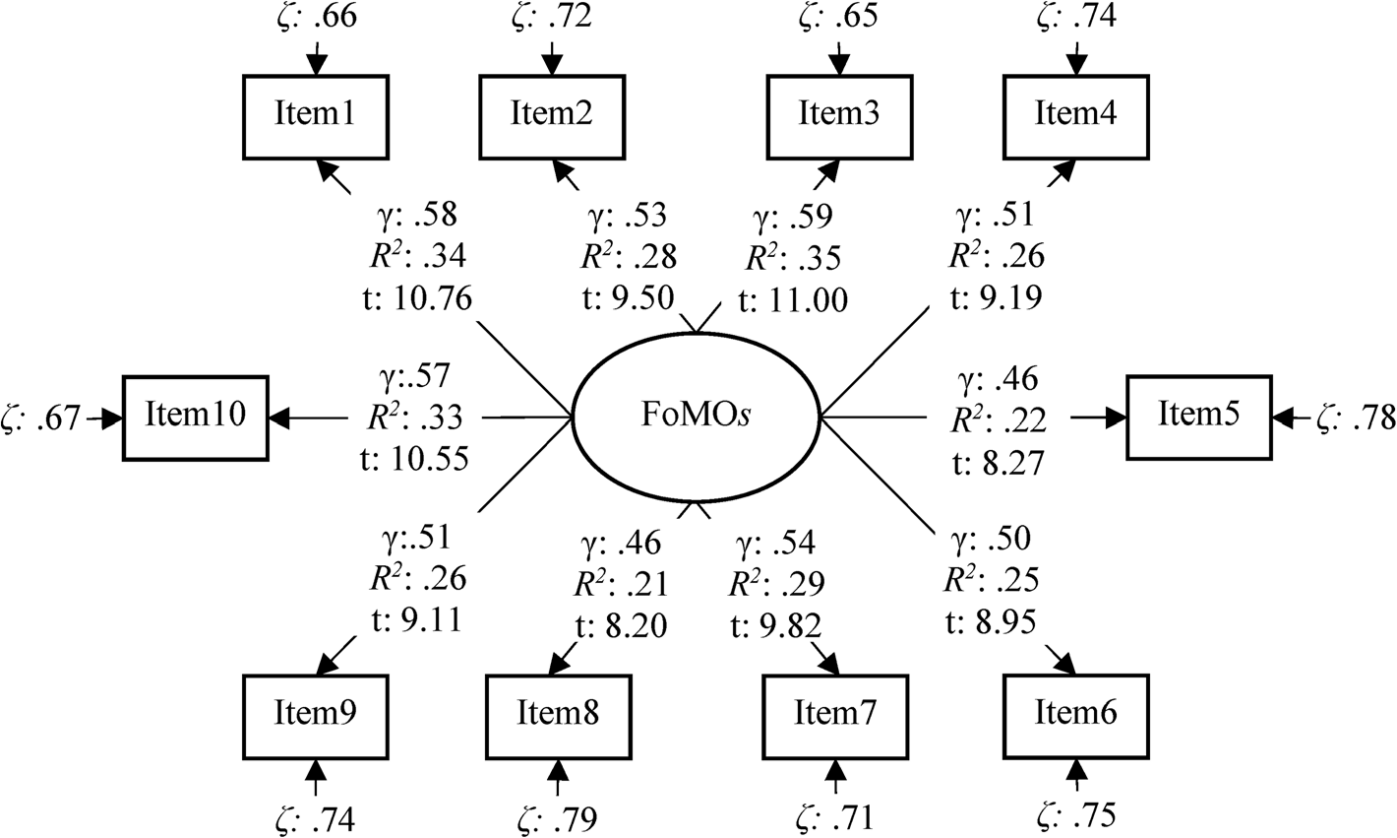
The standardized loadings, standard errors, *t*, and *R*^2^ values of the FoMOs. *N* = 354, γ = standardized loadings, ζ = error loading

The standardized loadings, standard errors, *t* values, and *R*^2^ values of the FoMOs are presented in Fig. 1.

As seen in Fig. 1, the factor loadings of the scale items ranged from .46 to .59. Additionally, all *t* values are significant. The model was found to fit the observed data well: *χ*^2^_(35, *N* = 354)_ = 93.75, *p* < .001; GFI = 0.95; CFI = 0.91; SRMR = 0.050; RMSEA = 0.069. The confirmatory factorial analysis (CFA) results for women [*χ*^2^_(35, *N* = 202)_ = 87.69, *p* < .001; GFI = 0.92; CFI = 0.87; SRMR = 0.064; RMSEA = 0.077] and men [*χ*^2^_(35, *N* = 152)_ = 62.63, *p* < .001; GFI = 0.92; CFI = 0.90; SRMR = 0.062; RMSEA = 0.072] samples also provided an acceptable fit to the data, although CFI value was slightly lower for women. In addition, *t* test for comparing Turkish FoMO scale scores of women and men indicated that there was no significant difference between the mean scores of women (*M* = 23.01, sd = 6.75) and men (*M* = 23.76, sd = 7.08; *t*_(352)_ = 1.02, *p* = .31 > .05). On the other hand, the one-way ANOVA results revealed that the FoMO scale scores differ significantly for the participants in terms of personal status (e.g., empployees, retired, students) (*F*_(2, 351)_ = 10.19, *p* < .001, η^2^ = .055). According to the tukey multiple comparison results, the FoMO scale scores of the students (*M* = 25.61, *SD* = 6.82) were significantly higher than those of the employees (*M* = 22.36, *SD* = 6.67) and retired participants (*M* = 21.46, *SD* = 6.69).

Finally, in this study, the measurement invariance (configural, metric, scaler and strict) across the groups of Facebook users and other social media users was examined. Measurement invariance was evaluated by GFI, CFI, ΔCFI, and RMSEA.

As can be seen in Table 1, the values of fit indices and *∆*CFIs are sufficient for providing configural and metric invariances, although the RMSEA values are slightly higher. On the contrary, the GFI for strict invariance, both the ΔCFI and GFI for scalar invariances are not sufficient.

**Table 1:** Fit indices for measurement invariances

Invariance	χ^2^	*df*	GFI	CFI	*∆*CFI	RMSEA
Configural invariance	152.83	70	.909	.937	–	.082
Metric invariance	170.62	79	.898	.928	.009	.081
Scalar invariance	214.805	98	.871	.905	.022	.082
Strick invariance	217.622	109	.865	.907	.002	.075

These findings show that the configural and metric invariances have been established across Facebook and other social media users, but the scalar and strict invariances have not.

## Study II

### The purpose

The study II aimed to investigate the concurrent validity of the Turkish version of FoMOs to provide additional evidence for the validity. In order to investigate the concurrent validity of the scale, the correlations between the Bergen Facebook Addiction Scale (BFAS), the Satisfaction with Life Scale (SWL), the time spent by participants in social media, and the Turkish FoMOs scores were calculated. It was expected that Turkish FoMOs would have positive correlations with the BFAS and average time spent on social media but negative correlation with the SWL.

### Method

#### Participants

The participants were 149 male and 222 female social media users at the ages ranging from 15 to 70 (M = 33.65, SD = 12.33), 59% of whom were employees, 11 retired, and 30 students. The participants’ preferred social media platforms were Facebook (65%), Whatsapp (22%), Twitter (8%), and Instagram (5%). The answer to the question of the most important reason why participants use social media demonstrated that 53.1% used the social media to keep track of social information, 25.9% to communicate with friends, and 21.0% to share ideas, opinions, and audio/visual material such as videos and pictures. The on-line surveying method was used during the data collection process. The same procedure about the recruitment of participants in study I was also carried out in study II.

### Measures

#### Bergen Facebook Addiction Scale (BFAS; Andreassen, Torsheim, Brunborg, & Pallesen, 2012)

BFAS was used to measure participants’ problematic facebook use levels. This scale consists of 18 items (e.g., “Become restless or troubled if you have been prohibited from using Facebook?”). Participants are asked to respond on a 5-point Likert scale (1 = very rarely and 5 = very often). Possible scores range from 18 to 90 with higher scores represent a higher level of problematic Facebook use. Turkish adaptation of this scale was carried out by Akin et al. (2013). The authors of the scale reported that the Turkish version of BFAS was found to well fit the data (*χ*^2^ = 291.88, df = 118, *p* < 0.001, RMSEA = .061, CFI = .95, GFI = .92, IFI = .95, and SRMR = .040) and has an acceptable internal consistency (Cronbach’s alpha = .93).

#### Satisfaction with Life Scale (SWLS; Diener, Emmons, Larsen, & Griffin, 1985)

SWLS was used to measure participants’ life satisfaction levels. This scale consists of five items (e.g., “In most ways my life is close to my ideal”). Participants are asked to respond on a 7-point Likert scale (1 = strongly disagree and 7 = strongly agree). Possible scores range from 5 to 35 with higher scores represent a higher level of life satisfaction. Turkish adaptation of this scale was carried out by Durak, Senol-Durak, and Gencoz (2010). Durak and colleagues reported that the Turkish version of the SWLS was well fit to the data (*χ*^2^/df = 2.026, IFI = .994, TLI = .987, CFI = .994, SRMR = .020, and RMSEA = .043) and has acceptable internal consistency (Cronbach’s alpha = .81).

### Procedures

As a first step, the association between FoMOs and problematic Facebook use, average time spent on social media, and life satisfaction (SWL) were examined. Pearson (*r*) correlation was used to obtain the correlation coefficients between the variables. Additionally, the descriptive data were also reported.

### Results

Table 2 illustrates the results of correlation analysis performed to assess the concurrent validity of Turkish FoMOs.

**Table 2:** Correlations and descriptive statistics of the study variables

Variable	Bivariate correlations				Descriptive Statistics		
	1	2	3	4	*M*	*SD*	Range
1. FoMOs	–				22.89	6.84	10–46
2. Problematic Facebook use	.43^**^	–			31.25	11.94	18–80
3. Life satisfaction	− .21^**^	− .15^**^	–		20.78	6.89	5–35
4. Average time^a^	.24^**^	.28^**^	− .05	–	191.44	160.08	5–780

**p < .01

^a^Time given as minute

The bivariate correlation tests revealed that the Turkish version of the FoMOs demonstrated a low positive correlation with BFAS (*r* = .43, 95% CI = .34, .51), but a little negative correlation with the SWLS (*r* = − .21, 95% CI = − .30, − .11). On the other hand, the relationship between the FoMO scores and the time spent as a minute in social media (*r* = .24, 95% CI = .14, .33) was also positive, even if it was at low level.

The mean FoMO score of the participants indicated that participants had medium level of the FoMO (*M* = 22.89, *SD* = 6.84). However, the means scores obtained from the BFAS (*M* = 31.25, *SD* = 11.94) and SWL (*M* = 20.78, *SD* = 6.89) scales were relatively low. The participants spent more than 3 h (*M* = 191.44_min_, SD = 160.08_min_) in social media on a typical day.

## Study III

### The purpose

The aim of study 3 was to investigate the internal consistency and temporal stability of the scale.

### Method

#### Participants

The reliability tests of the Turkish version of FoMOs were based on the three separate groups of participants. Both the participants in study I (57.1% females, 42.9% males, *M* = 32.97, *SD* = 13.04), and study II (59.8% females, 40.2% males, *M*_age_ = 33.64, *SD* = 12.33) were used in order to determine the internal consistency of the scale. We performed test re-test reliability over a 4-week interval with 61 participants (55.7% females, 44.3% males, *M*_age_ = 23.18, *SD* = 1.57) using Pearson’s *r* to analyze the associations between the test and re-test scores.

### Results

Table 3 presents the Cronbach’s alphas and test-retest correlation coefficients for the whole/entire study and for each study separately.

**Table 3:** Reliability results of the FoMOs

	Study I (*n* = 354)	Study II (*n* = 371)	Total data (*N* = 725)	Test-retest
FoMOs	.79	.78	.78	.86

Cronbach alpha coefficients were found as .79 for study I, .78 for study II, and .78 for total data. Four-week test-retest reliability was calculated as .86. These findings suggest that the Turkish version of the FoMOs has an acceptable reliability.

## Discussion

In this scale adaptation study, the factor structure of the Turkish version of the FoMOs developed by Przybylski et al. (2013) was examined via confirmatory factor analysis. Additionally, to determine the concurrent validity of the scale, the correlations between the FoMOs and (BFAS), (SWL), and the time spent in the social media were on the focus of the investigation.

The study has shown that the adapted version of the fear of missing out scale into Turkish context is a valid and reliable instrument for individuals aged between 15 and 72 years. The results of the CFA obtained from the present study yielded a unifactorial solution as in the original version and in line with previous adaptations of the original FoMO scale (Gökler et al., 2016; Al-Menayes, 2016; Perrone, 2013). According to goodness-of-fit indices, the model fits the data well similar to the previous adaptation studies mentioned above. Although the fit indices obtained from the CFA analysis conducted separately for men and women are slightly low, they still provide acceptable fits to the data for men and women samples.

In this study, the measurement invariance is supported at the level of configural and metric invariance, but not at the level of scalar and strict invariance. Thus, it is again safe to claim that Facebook and other social media users make use of the similar conceptual insights while responding to the scale item, and the relationship between the measured features (FoMO) and the FoMO items for Facebook and other social media users is also analogous.

The Cronbach alpha values found in study I (*α* = .79) and in study II (*α* = .78) are lower than the original version’s alpha value (α = .90) (Przybylski et al., 2013) and than the alpha values of Spanish (α = .85) and English (α = .91) versions of the scale. However, these values are within the acceptable levels and very close to the Cronbach alpha value of the first Turkish version of the scale (α = .81) by Gökler et al. (2016).

Although the relationships between the FoMOs and other scales used in this study, and the time spent in the social media is within the expected directions in terms of negative or positive relationship, their magnitudes are smaller than the expected values. However, the medium positive relationship between Turkish FoMOs and Bergen Facebook Addiction Scale (*r* = .43, *p* < .01) is not too far from than the relationship between FoMOs and Bergen Facebook Addiction Scale (*r =* .56, *p* < .01) found in the study by Tomczyk and Selmanagic-Lizde (2018). The weak negative relationship between Satisfaction with Life Scale and Turkish version of the FoMOs (*r* = − .21, *p* < .01) is also similar to the relationship between FoMO and overall life satisfaction (*r* = − .24, *p* < .01) as posited in the original study of scale of Przybylski et al. (2013). Additionally, the positive relationship between participants’ FOMO scores and the average time that they spent on social media as a daily routine (*r* = .24, *p* < .01) is another supportive finding for the satisfactory of concurrent validity of the Turkish version of the scale.

When the strength of the study is considered, Turkish FoMO scale can be used to measure for wider age groups, including students, employees, and retirees. However, this study has some limitations to be considered. The most important limitation of this study is related to its generalizability. Since the data gathered by the online surveying method, and the samples are non-probability samples, we confronted with a limited access to certain demographic groups that could effect generalizability of the scale’s findings negatively. For instance, the ratios of the retirees, students, Twitter, and Instagram users were too low when compared with other subgroups or populations in the samples such as the employees, Facebook, and Whatsapp users.

On the other hand, as in most studies where self-assessment reports are used, the possibility of some participants not being able to answer the scale meticulously in this study could also be seen as another limitation.

## Conclusion

Although being aware of the fact that there lie some the limitations as mentioned above, on the basis of findings of this study, the psychometric properties of the Turkish version of FoMO scale is satisfactory to measure the FoMO in a wide range of ages in the Turkish context.

## Abbreviations

ANOVA: Analysis of variance
BFAS: Bergen Facebook Addiction Scale
CFA: Confirmatory factorial analysis
CFI: Confirmatory Fit Index
FoMO: Fear of missing out
FoMOs: Fear of missing out scale
GFI: Goodness-of Fit Index
RMSEA: Root mean square error of approximation
SDT: Self-determination theory
SRMR: Standardized root-mean-square residual
SWL: Satisfaction with Life Scale
